# Ashwagandha‐Induced Herb‐Induced Liver Injury Assessed With the Updated 2016 RUCAM

**DOI:** 10.1155/crhe/8417352

**Published:** 2026-07-29

**Authors:** Samuel Pérez Pérez, Erika Zumaqué-Valverde, María Fernanda Saavedra Chacón, Ariel Antonio Arteta Cueto

**Affiliations:** ^1^ Gastrohepatology Research Group, University of Antioquia, Medellín, Antioquia, Colombia, udea.edu.co; ^2^ Departmet of Hepatology, Cancer Institute Las Americas, Auna, Medellin Antioquia, Colombia; ^3^ Department of Hepatology, CES Clinic, Medellín, Antioquia, Colombia; ^4^ Department of Pathology, University of Antioquia, Medellín, Antioquia, Colombia, udea.edu.co; ^5^ Pathology Research Group, University of Antioquia (GRIP-UdeA), Medellín, Antioquia, Colombia; ^6^ Pathology Laboratory, CES Clinic, Medellín, Antioquia, Colombia

**Keywords:** case reports, hepatotoxicity, herb-induced liver injury, *Withania somnifera*

## Abstract

Ashwagandha‐induced herb‐induced liver injury (HILI) has increasingly been reported; however, many published cases show methodological variability in causality assessment and limited clinicopathological correlation. We present a case of ashwagandha‐associated liver injury with systematic causality evaluation using the updated 2016 Roussel Uclaf Causality Assessment Method (RUCAM) and comprehensive clinicopathological correlation. A previously healthy 27‐year‐old woman developed jaundice and generalized pruritus after consuming ashwagandha for three days. Infectious, autoimmune, and metabolic etiologies were excluded. Abdominal magnetic resonance imaging showed no structural abnormalities, while liver biopsy demonstrated a classic cholestatic pattern without fibrosis. Causality assessment using the updated 2016 RUCAM yielded a score of 7 points, consistent with probable HILI. The patient received symptomatic management, corticosteroids, and azathioprine, with progressive clinical and biochemical improvement. This case highlights the value of a systematic causality approach using updated RUCAM criteria together with complete clinicopathological correlation to strengthen diagnostic confidence in suspected HILI associated with herbal supplements.

## 1. Introduction

Herb‐induced liver injury (HILI), including injury caused by herbal products and dietary supplements, has become an increasingly recognized cause of acute liver injury worldwide. Although liver injury related to exogenous agents has traditionally been associated with conventional medications, recent decades have seen a steady rise in cases linked to herbal and dietary supplements (HDSs) [1–4]. This growing trend represents an important clinical challenge given the widespread use of these products and the limited regulation surrounding their safety [4].

Among these supplements, *Withania somnifera* (ashwagandha), a plant commonly used in Ayurvedic medicine and now widely marketed worldwide as a “natural” supplement, has emerged as a potentially hepatotoxic agent [5, 6]. Ashwagandha is frequently promoted for its anxiolytic, adaptogenic, and immunomodulatory properties; however, its safety profile remains incompletely understood, and an increasing number of cases of liver injury associated with its use have been reported [2, 3, 5]. The clinical presentation of ashwagandha‐related hepatotoxicity is variable, ranging from hepatocellular injury to cholestatic or mixed patterns, commonly presenting with symptoms such as jaundice, pruritus, and fatigue [5, 6]. Although most reported cases improve after discontinuation of the supplement, severe presentations have also been described, particularly in patients with underlying liver disease [5].

The diagnosis of HILI remains particularly challenging because there are no specific biomarkers to confirm the condition. As a result, diagnosis relies largely on establishing a compatible temporal relationship between exposure and liver injury while carefully excluding alternative etiologies such as viral hepatitis, autoimmune liver disease, metabolic disorders, biliary obstruction, alcohol‐related liver disease, and exposure to other hepatotoxic agents [7]. Because of these limitations, structured causality assessment tools are strongly recommended. The updated 2016 Roussel Uclaf Causality Assessment Method (RUCAM) is currently considered the most widely accepted and validated tool for assessing causality in suspected cases of drug‐induced liver injury and HILI [7]. This method standardizes evaluation through objective criteria, including time to onset, course after withdrawal, risk factors, exclusion of alternative causes, known hepatotoxicity, and response to reexposure.

Given the increasing recognition of hepatotoxicity associated with herbal supplements and the need to strengthen the available evidence regarding their safety profile, we present the case of a young woman who developed ashwagandha‐induced liver injury, with causality assessed using the updated 2016 RUCAM [7].

## 2. Case Presentation

A 27‐year‐old woman with no relevant past medical history was admitted for five days (July 16–20, 2025) to a tertiary‐level referral hospital in Medellín (Antioquia, Colombia) with a 1‐week history of generalized pruritus, jaundice, and right upper quadrant pain. Viral hepatitis serologies for hepatitis A, B, and C were negative (Table 1). As part of the initial evaluation, a biliary ultrasound showed mild thickening of the gallbladder wall, without gallstones or sonographic signs of cholecystitis; no other relevant abnormalities were identified. A possible toxic or herb‐related liver injury was considered since all the other examinations were negative, the toxicology service recommended N‐acetylcysteine for 24 h. She was discharged with outpatient hepatology follow‐up and repeat liver function tests.

**TABLE 1:** External laboratory results.

Test date	ALT (U/L)	AST (U/L)	Total bilirubin (mg/dL)	Direct bilirubin (mg/dL)	HAV IgM	HBsAg	Anti‐HCV
July 15, 2025	913	324	2.67	—	Nonreactive	Nonreactive	Nonreactive
July 18, 2025	654	267	2.72	1.65	—	—	—
July 18, 2025	552	164	2.34	1.8	—	—	—
July 20, 2025	550	196	2.0	1.5	—	—	—
August 30, 2025	587	264	4.1	2.46	—	—	—

*Note:* ALT, alanine aminotransferase; AST, aspartate aminotransferase; HAV IgM, antibody to hepatitis A virus; HBsAg, hepatitis B surface antigen; anti‐HCV, antibody to hepatitis C virus.

At the follow‐up evaluation (August 30, 2025) (Table 1), transaminase elevation was again documented, prompting redirection of the patient to the emergency department for expanded studies. The patient was admitted to the emergency department of a quaternary care hospital in Medellín, Antioquia, where during the medical history, inquiry was made regarding a history of herbal supplement consumption, revealing that she had consumed a herbal supplement containing *Withania somnifera* (ashwagandha) for 3 days prior to symptom onset. Physical examination confirmed mucocutaneous jaundice without relevant abdominal findings.

Liver profile alteration with hepatocellular predominance was identified, with an R ratio of 16.8 according to the updated 2016 RUCAM classification for hepatocellular injury. Infectious etiologies were again ruled out, and autoimmune studies were negative (Tables 2 and 3). Causality assessment was performed using the updated 2016 RUCAM, yielding a total score of 7 points, consistent with a probable HILI secondary to ashwagandha exposure. Scoring included compatible time to onset, decrease in ALT after withdrawal, exclusion of alternative causes, and previously reported hepatotoxicity associated with ashwagandha. No concomitant hepatotoxic agents, relevant risk factors, or reexposure were identified. The detailed RUCAM assessment is summarized in Table 4.

**TABLE 2:** Initial laboratory results.

Laboratory test	Result	Units	Reference range
Hemoglobin	15.6	g/dL	(12.00–16.00)
Mean corpuscular volume	88	fL	(86.00–98.00)
White blood cells	9880	10^3^/μL	(4.50–11.00)
Neutrophils	4810	10^3^/μL	(1.40–6.50)
Lymphocytes	4170	10^3^/μL	(1.20–3.40)
Eosinophils	110	10^3^/μL	(0.045–0.44)
Platelets	337.000	10^3^/μL	(150.000–450.000)
ALT	952	U/L	(0–33.00)
AST	417	U/L	(0–32.00)
Alkaline phosphatase	178	U/L	(35.00–104.00)
GGT	30	U/L	(6.42)
Total bilirubin	4.0	mg/dL	(0.00–1.00)
Direct bilirubin	2.8	mg/dL	(0.00–0.20)
Creatinine	0.7	mg/dL	(0.51–0.95)
BUN	13	mg/dL	(6.00–20.00)
Prothrombin time (PT)	10.1	s	(9.73–11.66)
INR	0.95		(1.00–4.00)
aPTT	24	s	(22.50–35.00)
Anti‐HBc	1.960		Values > 1.00 considered negative; ≤ 1.00 considered positive
HBV antibodies	2.00	UI/L	< 10 IU/L: no immunity; ≥ 10 IU/L: immunity present
HAV IgM	0.34		Nonreactive: < 1.0; Reactive: ≥ 1.00

*Note:* ALT, alanine aminotransferase; AST, aspartate aminotransferase; HAV IgM, antibody to hepatitis A virus.

Abbreviations: aPTT, activated partial thromboplastin time; BUN, blood urea nitrogen; GGT, gamma‐glutamyl transferase; HBV, hepatitis B virus; INR, international normalized ratio; PT, prothrombin time.

**TABLE 3:** Autoimmune studies.

Test	Result	Units	Reference range
Serum IgG	1330.0	mg/dL	(700–1600)
Antimitochondrial antibody (IFA)	Negative		
Anti–smooth muscle antibody (IFA)	Negative		
Antinuclear antibodies (IFA)	Negative		

**TABLE 4:** Updated 2016 RUCAM assessment for hepatocellular herb‐induced liver injury associated with ashwagandha.

RUCAM domain	Patient findings	Score
Type of liver injury	Hepatocellular pattern based on R ratio ≥ 5. ALT: 952 U/L (ULN 33 U/L); ALP: 178 U/L (ULN 104 U/L). R ratio = 16.8	—
Time to onset from herbal product use	Symptoms developed approximately 3 days after initiation of ashwagandha consumption, compatible with suggestive chronology	+1
Course of ALT after cessation	Progressive decrease in transaminase levels after withdrawal of the herbal supplement	+2
Risk factors	No alcohol use; age < 55 years	0
Concomitant drugs/herbal products	No concomitant hepatotoxic agents identified	0
Exclusion of nonherbal causes	Negative serologies for hepatitis A, B, and C; negative autoimmune panel (ANA, ASMA, AMA); MRI without biliary obstruction; no metabolic disease identified on histopathology	+2
Previous hepatotoxicity of implicated herb	Ashwagandha‐associated hepatotoxicity previously reported in the scientific literature	+2
Reexposure	Not documented	0
Total score		7 points
RUCAM 2016 causality grading		Probable HILI

*Note:* ALT, alanine aminotransferase; ALP, alkaline phosphatase.

Abbreviations: HILI, herb‐induced liver injury; RUCAM, Roussel Uclaf Causality Assessment Method; ULN, upper limit of normal.

**FIGURE 1:**
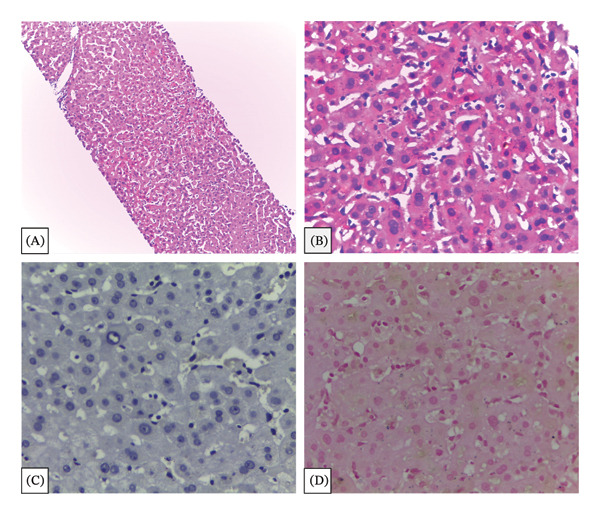
Liver biopsy. The characteristics of the liver biopsy showed a scarce portal and lobular inflammatory component, highlighting the presence of cholestasis ((A) hematoxylin and eosin 100X). Intracytoplasmic cholestasis accompanied by the presence of bile plugs ((B) hematoxylin and eosin 400X). Copper ((C) 400X) and iron ((D) 400X) stains were negative.

Further diagnostic evaluation included contrast‐enhanced abdominal MRI and liver biopsy. The image showed no structural abnormalities or signs of obstructive cholestasis. Liver biopsy revealed a classic cholestatic pattern with preservation of hepatic architecture and absence of fibrosis, findings compatible with HILI (Figure 1).

In the histopathological study, immunohistochemistry showed positivity for cytokeratin 7 (CK7) in the bile ducts of most portal spaces, while the CD56 marker was negative. Special stains for iron and copper showed no deposits, ruling out metabolic diseases. Trichrome staining demonstrated no pathological collagen deposits, with a fibrosis stage of 0/4, and reticulin staining showed preservation of the reticular fiber framework. Together, these findings allowed to rule out primary biliary disease, metabolic pathologies, and the presence of fibrosis and showed a highly suggestive cholestatic pattern in the clinical context of HILI.

**FIGURE 2:**
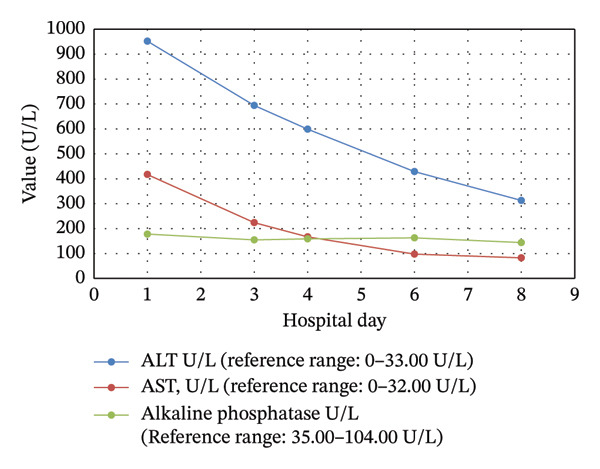
Evolution of ALT, AST, and alkaline phosphatase during hospitalization.

**FIGURE 3:**
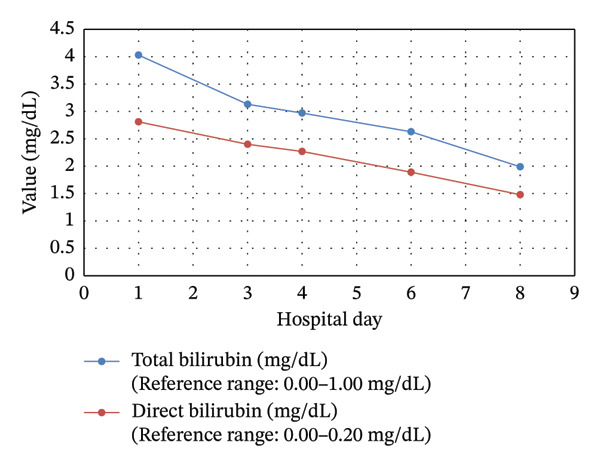
Evolution of total and direct bilirubin during hospitalization.

Initial symptomatic management included cholestyramine for pruritus control and ursodeoxycholic acid in the setting of mild cholestatic features. Due to persistent and refractory pruritus, a multimodal therapeutic approach was implemented, including gabapentin, sertraline, and fenofibrate, which was well tolerated and resulted in clinical improvement. Despite symptomatic benefit, liver function tests continued to show persistent transaminase elevation compared with previous external studies (Table 1). Consequently, systemic corticosteroid therapy was initiated, leading to progressive biochemical improvement (Figures 2 and 3). The decision to start corticosteroids was individualized because of persistent biochemical abnormalities and severe refractory symptoms, although evidence supporting the routine use of immunosuppression in HILI remains controversial. Subsequently, azathioprine was introduced as a steroid‐sparing agent to facilitate a more rapid outpatient corticosteroid taper.

The integration of clinical, biochemical, and histological findings, together with systematic exclusion of other etiologies, was compatible with the diagnosis of ashwagandha‐induced liver injury. The patient showed progressive clinical and biochemical improvement after supplement discontinuation and initiation of immunomodulatory treatment.

## 3. Discussion

Hepatotoxicity induced by herbal supplements (HILI) has been increasingly recognized as a relevant cause of acute liver damage [4]. Among these products, ashwagandha (*Withania somnifera*) has emerged as a consistently documented hepatotoxic agent in case reports and clinical series globally. Available evidence regarding ashwagandha‐associated hepatotoxicity has demonstrated a relatively consistent clinical pattern, although with variability in severity and presentation. Early reports predominantly described cholestatic manifestations characterized by marked jaundice and pruritus. Inagaki et al. reported a case of severe and prolonged intrahepatic cholestasis following consumption of a pure ashwagandha supplement, with liver biopsy demonstrating canalicular cholestasis and favorable evolution after product withdrawal and treatment with ursodeoxycholic acid [6]. Similarly, Zhuang et al. documented a case with short latency, biopsy findings compatible with mild lobular cholestasis, and progressive biochemical recovery after discontinuation of the supplement [8].

Taken together, these reports suggest that jaundice, pruritus, and cholestatic histological findings represent recurrent characteristics of ashwagandha‐associated hepatotoxicity. Our case shares multiple features with these previous reports, particularly the presence of marked pruritus, jaundice, and a predominantly cholestatic histopathological pattern. However, despite the hepatocellular biochemical classification according to the R ratio, liver biopsy demonstrated a cholestatic pattern. This variability across clinical and histopathological findings may reflect the heterogeneous nature of ashwagandha‐associated liver injury [5, 8].

The multicenter series published by Philips et al. considerably expanded the clinical spectrum of this entity by including patients with underlying chronic liver disease, some of whom progressed to fatal acute liver failure [5]. This finding is particularly relevant because it suggests that individual susceptibility and hepatic functional reserve may play a determinant role in injury severity. In addition, the histological findings described by Philips et al., including prominent canalicular cholestasis, hepatocellular necrosis, and portal inflammatory infiltrates with eosinophils, showed remarkable correlation with the patterns observed in other reports and support the existence of an immune‐mediated or idiosyncratic injury associated with the supplement.

Other authors, including Tóth et al., Lubarska et al., and Suryawanshi et al., also documented variable clinical presentations ranging from self‐limited liver injury to acute liver failure requiring liver transplantation [1, 8, 9]. The progression to severe forms observed in some patients contrasts with the widespread perception of safety associated with herbal products and highlights the clinical importance of recognizing ashwagandha‐associated HILI as a potentially severe entity [10].

Nevertheless, although the clinical consistency across different reports strengthens the association between ashwagandha and hepatotoxicity, the interpretation of the available evidence is limited by methodological heterogeneity in causality assessment. Many of the earlier cases were published before the widespread adoption of the updated 2016 RUCAM or used alternative methods based on clinical judgment and local assessment systems, resulting in methodological heterogeneity across published reports [1, 5, 6, 9]. Since HILI remains a diagnosis of exclusion and lacks specific biomarkers, the absence of a structured causality assessment may affect the accuracy of causal probability estimation and limit comparability across studies, particularly in the presence of confounding factors or concomitant exposure to other products. In this context, the systematic implementation of the updated RUCAM represents an important advance, as it allows objective integration of exposure chronology, clinical and biochemical evolution following withdrawal of the suspected agent, and exclusion of alternative etiologies, thereby improving diagnostic consistency and reproducibility across reports.

From a pathophysiological perspective, the variability observed among different cases likely reflects complex and multifactorial mechanisms. Siddiqui et al. demonstrated that withanone, one of the principal withanolides present in ashwagandha, can form stable covalent adducts with DNA and generate cellular oxidative stress [11, 12]. Although glutathione‐dependent antioxidant systems may partially neutralize these metabolites, conditions of individual susceptibility or preexisting liver disease could favor the development of hepatotoxicity and explain both the clinical heterogeneity and the greater severity observed in some patients.

In conclusion, ashwagandha constitutes a potential causative agent of HILI, with a variable clinical spectrum ranging from self‐limited cholestatic forms to severe cases of acute liver failure requiring liver transplantation. Reports published to date demonstrate relatively consistent clinical, biochemical, and histopathological patterns, particularly the presence of jaundice, pruritus, and cholestatic findings on liver biopsy, which strengthens the association between this herbal supplement and hepatotoxicity. However, much of the available evidence presents methodological heterogeneity in causality assessment, partially limiting the comparability and strength of some reports. In this context, the systematic application of the updated 2016 RUCAM represents an important tool to strengthen causality assessment, improve methodological consistency, and consolidate a more robust body of evidence regarding the hepatotoxic profile of ashwagandha and other herbal supplements.

## Funding

This research did not receive any specific grant from funding agencies in the public, commercial, or not‐for‐profit sectors.

## Consent

Written informed consent was obtained from the patient for publication of this clinical case and corresponding diagnostic images. The patient expressly authorized the use of information for scientific and academic dissemination purposes, ensuring at all times the confidentiality and anonymity of her personal data.

## Conflicts of Interest

The authors declare no conflicts of interest.

## Data Availability

The data supporting the findings of this case report are available from the corresponding author upon reasonable request. All relevant data are included within the article; however, additional data are not publicly available due to privacy and ethical restrictions.
